# Relationships between executive functioning and self‐concept in children referred for neuropsychological group rehabilitation and typically developing children

**DOI:** 10.1111/jnp.70029

**Published:** 2026-01-14

**Authors:** Elina Vierikko, Heini Saarimäki, Kati Rantanen

**Affiliations:** ^1^ Department of Psychology, Faculty of Social Sciences University of Tampere Tampere Finland; ^2^ Department of Rehabilitation and Psychosocial Support Tampere University Hospital Tampere Finland; ^3^ Department of Psychology, Faculty of Medicine University of Helsinki Helsinki Finland

**Keywords:** children, executive functioning, neuropsychological group intervention, self‐concept

## Abstract

This study examines the relationship between executive functions (EFs) and self‐concept (SC) in children referred for neuropsychological group rehabilitation due to EF deficits, compared with typically developing (TD) children. EFs and SC are vital for academic performance and social interactions, yet the link between them in clinical populations remains underexplored. The study involved 42 children aged 7–13 with confirmed EF deficits and 104 TD children. EF was assessed using the Behavior Rating Inventory of Executive Functioning (BRIEF), while SC was measured with the Piers‐Harris Self‐Concept Scale for Children (P‐H2). Statistical analyses, including Mann–Whitney tests, Spearman correlations and hierarchical regression, were conducted to explore group differences and associations between EF and SC. Results indicated that children referred for EF intervention exhibited significantly lower SC across all domains compared with TD peers. Despite EF difficulties generally correlating with negative SC, significant associations were primarily observed in TD children, particularly in metacognition‐related domains. Gender and age influenced SC, with girls showing more positive SC than boys, and older children experiencing more SC challenges. Learning support levels also impacted SC, with intensified support linked to more negative SC. In conclusion, EF deficits are associated with negative SC in school‐aged children, with a more pronounced relationship in TD children than those referred for intervention. The study highlights the importance of considering individual variation in SC among children with EF challenges and suggests that future research should explore developmental trajectories and mediators between EF and SC, emphasizing tailored interventions to enhance positive SC in clinical populations.

## INTRODUCTION

Executive functions (EFs) and self‐concept (SC) are critical for school‐aged children's academic performance and social interactions. Although their interrelation has been widely studied in typically developing (TD) children, evidence from clinical populations with pronounced EF deficits remains limited. This study addresses this gap by examining the association between EF and SC in children referred for neuropsychological rehabilitation due to EF and attention deficits, and by comparing them with TD peers.

EFs refer to higher‐level cognitive processes necessary for goal‐directed behaviour (Welsh et al., 1991; Willcutt et al., 2005). Inhibition, cognitive flexibility and working memory (WM) are considered distinct but interacting core components of EF (Miyake et al., 2000). Inhibition refers to abilities to suppress automatic responses, cognitive flexibility (or shifting) entails switching attention between tasks or mental frameworks, and WM involves updating, monitoring and coding relevant information and replacing irrelevant or old information to maintain goal‐directed behaviour. These core components develop interactively during early childhood (Klenberg et al., 2001; Roberts Jr & Pennington, 1996) and form the basis for the more complex EF components, such as planning, problem‐solving and strategy use, which develop later in mid‐childhood and adolescence (Diamond, 2013; Huizinga et al., 2006; Lehto et al., 2003).

EF deficits are prevalent in paediatric developmental and neuropsychiatric disorders, such as attention deficit hyperactivity disorder (ADHD, Miklós et al., 2019; Tamm et al., 2021; Townes et al., 2023). These deficits or delays in EF development impact a child's ability to meet real‐world demands, often manifesting as self‐regulation challenges that lead to increased behavioural and emotional difficulties (Lonigan et al., 2017; Miyake & Friedman, 2012). Consequently, these difficulties adversely affect academic performance (Cortés Pascual et al., 2019; Spiegel et al., 2021) and peer relations (Holmes et al., 2016; Wang & Feng, 2024) and potentially lead to the development of a negative SC through repeated failures and negative feedback (Capelatto et al., 2014; Kita & Inoue, 2017).

SC refers to an individual's perception of their own characteristics, competencies and self‐worth, structured hierarchically across domains like academic, social, emotional and physical self‐concept (Harter, 2012; Marsh & O'Mara, 2008; Marsh & Shavelson, 1985). In childhood, SC is shaped by internal evaluations and external feedback, particularly from academic performance and peer interactions (Dapp & Roebers, 2021). EF, a crucial aspect of cognitive development, is essential for monitoring actions, interpreting feedback and adjusting behaviour, all of which are central to the formation and maintenance of SC. Impaired EF can compromise the ability to accurately assess one's behaviour and performance (Fogel, 2022), as seen in children with ADHD (Fisher et al., 2022; Hoza et al., 2002, 2004). This can lead to negatively biased self‐perceptions or overly positive self‐views, such as the positive illusory bias observed in some children with ADHD (Hoza et al., 2002, 2004).

In TD children, significant gender and age differences in SC have been observed during middle childhood. As a result of a complex interaction between cognitive abilities, academic achievement, environmental expectations and social competence, the global SC usually declines during middle childhood compared with early childhood (Harter, 2012; Marsh, 1989). Jacobs et al. (2002) found that boys felt more competent in sports and math, while girls felt more competent in language and arts. Cole et al. (2001) discovered that mean levels in self‐perceived academic competence, social acceptance and sports competence increased similarly across genders, while self‐perceived behavioural conduct declined. Girls perceived themselves as better behaved than boys. Unlike the study by Cole et al. (2001), no gender differences were observed in academic SC, likely because the questionnaire focused on global academic SC rather than specific areas like math or reading/writing where gender differences had previously been noted. Self‐perceived physical appearance decreased for females and increased for males. The transition from elementary to middle school was associated with destabilization in most self‐concept domains, a period marked by pubertal, cognitive and educational changes. Early‐onset gender differences have been found to persist over time (Jacobs et al., 2002; Wigfield et al., 1997), which are partly attributed to the different expectations and demands that adults place on both girls and boys.

Empirical findings support the assumption that SC and EF are interrelated in TD children. Pittari and Brown (2020) showed that SC and self‐esteem predicted parent‐rated EF in a small sample of 20 TD children aged 8 to 12, with SC explaining a substantial proportion of the variance in overall EF and the EF subdomains of emotional and cognitive regulation. Notably, SC related to intellectual and school status was the strongest predictor of cognitive regulation, highlighting the importance of academic self‐perceptions in EF. Also, Roebers et al. (2012) found that EF was significantly related to metacognitive control and SC, both cross‐sectionally and longitudinally, in an explorative study involving 209 elementary school children attending 1st grade. Individual differences in EF and metacognitive control were linked to academic outcomes, suggesting that these cognitive processes play a role in shaping self‐perceptions related to academic competence.

Only a few studies have simultaneously explored the relationships between SC and EF in clinical paediatric populations characterized by executive dysfunction. Similar associations have been observed in such populations. Bailey et al. (2018) reported greater EF difficulties linked to lower academic SC in 122 children and adolescents aged 8–17 referred for neuropsychological assessment. Interestingly, their study did not find direct associations between EF or SC and academic achievement, which was attributed to methodological differences in EF assessment—specifically, parent‐reported everyday EF (e.g. BRIEF, Gioia et al., 2000) versus performance‐based tasks used in earlier studies (see also Vriezen & Pigott, 2002). These findings suggest that children's self‐perceptions may be more closely tied to everyday EF challenges than standardized academic outcomes.

The reciprocal effects' model proposed by Marsh and Craven (2006) emphasizes the bidirectional relationship between SC and academic achievement, but the role of EF within this dynamic remains underexplored. Children with EF deficits and special needs, such as ADHD, learning difficulties or those receiving learning support, face challenges in academic performance, which may negatively affect their SC and self‐esteem (Harpin et al., 2016; Savolainen et al., 2018). However, treated ADHD is associated with better long‐term outcomes for SC and social function compared with untreated ADHD (Harpin et al., 2016). In addition, some children with ADHD exhibit positive illusory bias, overestimating their abilities in academic and social domains (Owens et al., 2007). While this bias may serve a self‐protective function and preserve motivation (McQuade et al., 2017), it can hinder self‐awareness and responsiveness to feedback (Bailey et al., 2018). However, an investigation into self‐regulatory skills revealed that children with ADHD rated themselves as more dysfunctional compared with TD peers, despite their self‐ratings not significantly diverging from adult evaluations (Rizzo et al., 2010). The findings indicate that self‐perception is not uniformly positive and can vary significantly among children with ADHD. Although EF deficits are well‐documented characteristics in children diagnosed with ADHD, only about 50% of these children have been identified as having EF deficits at the individual level, suggesting variability in impairment in this population (Lambek et al., 2011). This variability could also explain why findings regarding biased self‐perception are inconsistent and why not all children with ADHD have a positive illusory bias.

Despite growing interest in the interplay between EF and SC, few studies have concurrently explored these constructs in children with clinically significant EF deficits and TD children. Current evidence suggests that EF deficits may influence children's self‐perception, particularly in academic and regulatory domains. However, further research is needed to clarify these associations across various developmental profiles. Previous studies have largely focused on academic SC, often dividing it into specific areas related to mathematics and literacy. For example, Bailey et al. (2018) highlighted the need for more research on overall SC and other SC domains, as their study, like many others, primarily focused on academic SC. In a recent meta‐analysis, Betancourt et al. (2024) examined self‐esteem levels in children and adolescents with ADHD in comparison with their peers without the disorder, focusing on both global and domain‐specific self‐esteem, including academic, social and behavioural aspects. Their findings indicate that while children and adolescents with ADHD experience moderate impairments in self‐esteem, they do not uniformly perceive themselves negatively across all domains. This suggests the existence of factors that may enhance their global self‐esteem, potentially in areas that are not directly associated with ADHD.

There is a need to examine associations between EF and different SC domains, including global SC. The present study addresses this gap by investigating SC and its relationship with EF in two groups: children with EF deficits referred for a structured neuropsychological group rehabilitation programme, Rehabilitation of EXecutive Function and Attention (EXAT, Rantanen et al., 2018), and TD peers. By comparing these groups, the study aims to better understand how EF challenges may shape children's self‐perceptions. The study is part of a larger clinical intervention registry study at the Psychology Clinic of the Tampere University in Finland, which was assessed and approved by both the Ethics Committee of University of Tampere and the Tampere University Hospital, Pirkanmaa Hospital District.

The objectives are as follows:

1. To explore the differences in total SC and its domains between the EXAT and TD groups. Additionally, the impact of age, gender and learning support on SC was examined.
2. To assess how SC and its various domains (such as academic, social and emotional) relate to parent‐rated EF in school‐aged children.
3. To study the group differences (EXAT and TD groups, age and gender) in the association between SC and EF, aiming to identify whether EF deficits have a stronger link to negative SC in children undergoing neuropsychological intervention.

Based on earlier studies (Bailey et al., 2018; Betancourt et al., 2024; Capelatto et al., 2014; Pittari & Brown, 2020), it is anticipated that children with EF difficulties will report lower SC levels, particularly in academic and emotional domains. The hypothesis posits that the link between EF deficits and SC will be stronger in children referred for neuropsychological intervention due to EF deficits or ADHD, compared with TD children. This reflects the assumption that clinically significant EF problems have a more profound impact on self‐perception (Bailey et al., 2018; Capelatto et al., 2014; Pittari & Brown, 2020). Additionally, specific EF components related to cognitive and emotional regulation are expected to have varying associations with SC domains. For example, difficulties in cognitive regulation are predicted to be more closely connected to academic SC, while emotional regulation may be more related to emotional or social SC (McQuade et al., 2017; Pittari & Brown, 2020).

SC was expected to become more negative with age (Harter, 2012; Marsh, 1989). No significant gender differences were expected in academic SC, as the measure employed in this study assessed global academic SC rather than the domain‐specific areas of reading/writing and mathematics, in which gender differences have been reported in previous research.

## MATERIALS AND METHODS

### Participants

The clinical sample (EXAT group) comprised children referred for multilevel Rehabilitation of Executive Function and Attention (EXAT, Rantanen et al., 2018) intervention due to confirmed EF deficits at the Psychology Clinic, University of Tampere, Finland. EXAT is a manualized neuropsychological programme that combines cognitive EF training, behavioural strategies, social reinforcement and psychoeducation to enhance self‐regulation. The programme includes children's groups, parent sessions and teacher consultations and typically lasts about 9 months. Thus, the participants were not originally recruited for research purposes. Between 2014 and 2017, 106 children aged 6–13 were referred for EXAT rehabilitation, and their guardians provided informed consent to participate in the study. Inclusion criteria of this study were age between 6 and 13 years, confirmed deficits in EF and attention assessed at referral by local psychologists, and initiation of the first EXAT rehabilitation period, with no concurrent interventions or therapies. Exclusion criteria included developmental disabilities and severe psychiatric disorders (e.g. depression and anxiety), as well as incomplete data (missing questionnaires). As a result, 42 (54%) met the inclusion criteria and had complete behavioural questionnaire data available (Table 1). All participants of the EXAT group had prominent EF deficits at referral based on clinical neuropsychological assessments conducted by local psychologists with interviews, standardized tests (WPPSI‐III, Wechsler, 2009; WISC‐IV, Wechsler, 2010; NEPSY II, Korkman et al., 1997) and/or behavioural ratings (5 to 15, Kadesjö et al., 2004; Lambek & Trillingsgaard, 2015; and/or Attex, Klenberg et al., 2010). Means for both Full Scale IQ and Perceptual Reasoning Index were within normal range. Approximately 36% of participants had an ADHD diagnosis (ICD‐10; World Health Organization, 1992), and 40% were receiving ADHD medication (Table 1). In addition, five children had other diagnoses, including neurodevelopmental disorders (autism spectrum disorder and mixed disorder of scholastic skills), neurological disorder (epilepsy and cerebral palsy) and fetal alcohol syndrome.

**TABLE 1:** Demographic and medical background data of the EXAT* group and the TD (typically developing) group.

	EXAT group^a^ (*n* = 42)	TD group (*n* = 104)	Group comparison
Gender (boy/girl)	36/6	58/46	*χ* ^2^ (1) = 10.43, *p* = .001
Age, mean (SD)	9.5 (1.2)	9.8 (1.7)	*t*(106) = −0.81, *p =* .420
Mother's age, mean (SD)	39.6 (5.9)	40.0 (5.3)	*t*(67) = −0.33, *p* = .743
Father's age, mean (SD)	41.8 (7.5)	42.4 (6.2)	*t*(64) = −0.41, *p* = .682
Mother's education			*χ* ^2^ (2) = 9.40, *p* = .009
9–12 years	17	17	
12–15 years	8	25	
16 or more	17	60	
Unknown	0	2	
Father's education			*χ* ^2^ (2) = 5.32, *p* = .070
9–12 years	21	39	
12–15 years	2	20	
16 or more	18	43	
Unknown	1	2	
Diagnosis (ICD‐10)			Fisher *p* < .0001
No	21	104	
Yes (ADHD)	15	0	
Yes (other^b^)	5	0	
Medication for ADHD			Fisher *p* < .0001
No	25	104	
Yes	17	0	
Learning support at school			Fisher *p* < .0001
General support	7	96	
Intensified support	9	8	
Special support	26	0	
Full Scale IQ Mean (SD)	90.66 (16.04)		
Perceptual Reasoning Index Mean (SD)	98.56 (17.0)		
Conners' Parent Rating Scale‐R (T‐score)			
DSM‐IV Inattention Md (range)	63.0 (49.9–90)	47.0 (40.0–88.0)	*W* = 2817, *p* < .0001
DSM‐IV Hyperactivity‐Impulsivity Md (range)	61.0 (44.0–90)	46.0 (41.0–83)	*W* = 2724.5, *p* < .0001
DSM‐IV Total Md (range)	63.0 (47.0–85)	48.0 (40.0–89.9)	*W* = 2886.5, *p* < .0001

^a^ Comprised of participants referred for neuropsychological intervention.

^b^ Other neurodevelopmental disorder (ICD–10 codes F80–F84.5, F91.3), neurological disorder (epilepsy) or congenital malformation (fetal alcohol syndrome).

TD sample was drawn from a larger sample (*n* = 234, 6–15 years) of children attending mainstream elementary schools, based on their age (7–13 years), and parental and self‐ratings completed and returned. Two participants were excluded based on ADHD diagnosis and/or psychostimulant medication use. The resulting TD group consisted of 104 children (58 boys). None of the TD participants had a diagnosis of ADHD or any other neurological, developmental or psychiatric diagnoses or medication for ADHD symptoms. However, eight TD children had mild learning difficulties and received general learning support, such as remedial instruction as a part of general learning support. This is consistent with the Finnish educational system, where all children are entitled to general learning support regardless of medical diagnosis (Basic Education Act, 1998). Learning support is organized into three tiers: general, intensified and special support. General support includes universal strategies and resources within the regular classroom, such as differentiated instruction, remedial teaching and classroom accommodations. If these measures prove insufficient, intensified support is provided based on a pedagogical assessment. Intensified support is typically temporary and involves tailored learning plans, small‐group instruction and closer progress monitoring. Special support is intended for children with persistent learning difficulties and includes individualized education plans (IEPs), specialized teaching methods and additional resources, such as special education teachers or assistive technology. Importantly, children may receive intensified or special support based on educational needs alone, without a formal diagnosis.

### Measures

The demographic and medical background data for the EXAT group were obtained from the Psychology Clinic records based on structured interviews with the parents and information given at referrals by local psychologists, and from questionnaires (Table 1). Parents of both the EXAT and the TD groups completed a background information form along with the Conners' Parent Rating Scales–Revised (CPRS‐R; Conners, 1997). The CPRS‐R was utilized to assess participants' attention and a broad spectrum of behavioural problems prior to the intervention. This scale comprises 80 items, rated on a four‐point Likert scale (0 = not true at all or never, seldom; 3 = very much true or very often, very frequently). Specifically, the DSM‐IV symptom subscales for Inattention, Hyperactivity‐Impulsivity and Total score were employed in this study. Composite scores from the Conners' scales were individually transformed into T‐scores (*M* = 50, SD = 10), where higher T‐scores denote a greater level of behavioural problems. A T‐score of 65 or above (1.5 SD) was considered indicative of clinical‐level problems. The reliability of the scales was confirmed with Cronbach's alpha values of 0.935 for DSM‐IV Inattention, 0.863 for DSM‐IV Hyperactivity‐Impulsivity, and 0.933 for DSM‐IV Total score.

The use of the CPRS‐R as a background variable was crucial in establishing the baseline behavioural and attentional profiles of participants. For the TD group, it ensured that these children did not exhibit significant EF, attentional or behavioural difficulties, nor were they undergoing therapies related to neurodevelopmental concerns. This differentiation is essential for accurately comparing outcomes between the EXAT group and control group, thereby validating the study's findings and ensuring that any observed effects of the intervention are not confounded by pre‐existing conditions.

EFs were assessed using the parent report form of the Behavior Rating Inventory of Executive Functioning (BRIEF; Gioia et al., 2000). The parent form of BRIEF is comprised of 86 items rated on a 3‐point Likert scale (1 = *never*, 2 = *sometimes*, 3 = *often*), measuring eight subdomains of EF. The subscales of Inhibit, Shift and Emotional Control form a composite summary score of the Behavioral Regulation Index (BRI), and the subscales of Initiate, Working Memory, Plan/Organize, Organization of Materials, and Monitor form a composite summary score of the Metacognition Index (MI). The BRI and MI can further be combined to form a Global Executive Composite (GEC). Furthermore, two validity scales are included: Inconsistency and Negativity. The raw composite scores of each subscale and index were converted into norm‐referenced, age‐based T‐scores with a mean of 50 and a standard deviation of 10. Higher T‐scores indicate a higher level of EF problems, and the cut‐off level for the clinical range of problems is a T‐score > 65 (1.5 SD). The Cronbach's alpha for the BRIEF subscales and indexes ranged from 0.822 (Shift) to 0.928 (Emotional Control).

SC was measured with the Piers‐Harris Self‐Concept Scale for Children (P‐H2; Piers & Herzberg, 2002). P‐H2 is a self‐report consisting of 60 items, which are rated either 1 or 0. Score 1 denotes a positive attitude towards self and 0 a negative attitude. The P–H2 provides a Total Score (TOT) ranging from 0 to 60 points and reflecting a general self‐concept, and six subdomain scores: Behavioral Adjustment, Intellectual and School Status, Physical Appearance and Attributes, Freedom from Anxiety, Popularity, and Happiness and Satisfaction. The raw scores on the Total Score and the subdomain scales were converted to T‐scores (*M* = 50, SD = 10), with a normal range between 40 and 60 for the total score, and between 40 and 55 for the subdomains. Higher T‐scores represent a more positive attitude towards self. The P‐H2 has demonstrated strong reliability and internal consistency across various demographic groups. This includes both a normative sample from the United States (Piers & Herzberg, 2002) and a representative cohort from Ireland (Guerin & Tatlow‐Golden, 2019), with a Cronbach's alpha of .91 for the Total Scale and values ranging from .72 to .83 for the subscales. In the present sample, the Cronbach's alpha for the total score was 0.907, and for the six subdomains it ranged from 0.531 (Physical Appearance and Attributes) to 0.923 (Freedom from Anxiety). All questionnaires in this study were administered at the start of the rehabilitation, and only these baseline responses were used for analyses.

### Statistical analysis

IBM SPSS Statistics software (version 23.0, IBM Corp, 2015) and R (version 4.3.1, R Core Team, 2023) and RStudio (version 2025.05.1, Posit Software, PBC, 2025) were used for the statistical analyses. Group differences between EXAT group and TD group in EF and SC were examined with T‐scores of the P‐H2 and BRIEF subscales using Mann–Whitney tests. P‐values less than 0.05 were considered statistically significant. The effect size for group comparisons was calculated from the formula: $r=\frac{Z}{\sqrt{N}}$ (Fritz et al., 2012) using *wilcox_effsize* from *rstatix* package.

The relationship between EF and SC was examined with correlation tests and linear regression. We first investigated the association between each of the EF composite scores and SC total score and subdomain scores in all participants using Spearman correlation. Next, we examined whether this association is also present when controlling for age and gender differences between groups (e.g. *self‐concept ~ age + gender + group*). Finally, we investigated group differences in the association between EF and SC subscales using hierarchical linear regression, where steps included adding (1) group, (2) EF subscales (BRI and MI), and (3) group × EF interaction (e.g. *self‐concept ~* group * *EF_BRI*). The fit of the linear models was compared with *Anova* function and results were visualized with *ggplot2* package.

## RESULTS

### Group differences in SC and EF

There were more boys in the EXAT group than in the TD group (Table 1). Participants in the EXAT group also more often required learning support at school compared with participants in the TD group. Only eight participants in the TD group received intensified learning support due to mild learning or behavioural difficulties, whereas most children in the EXAT group received either intensified or special learning support. The groups did not differ in terms of the child's age, mother's and father's ages, or father's education. However, the mother's education was higher in the TD group than in the EXAT group.

**FIGURE 1:**
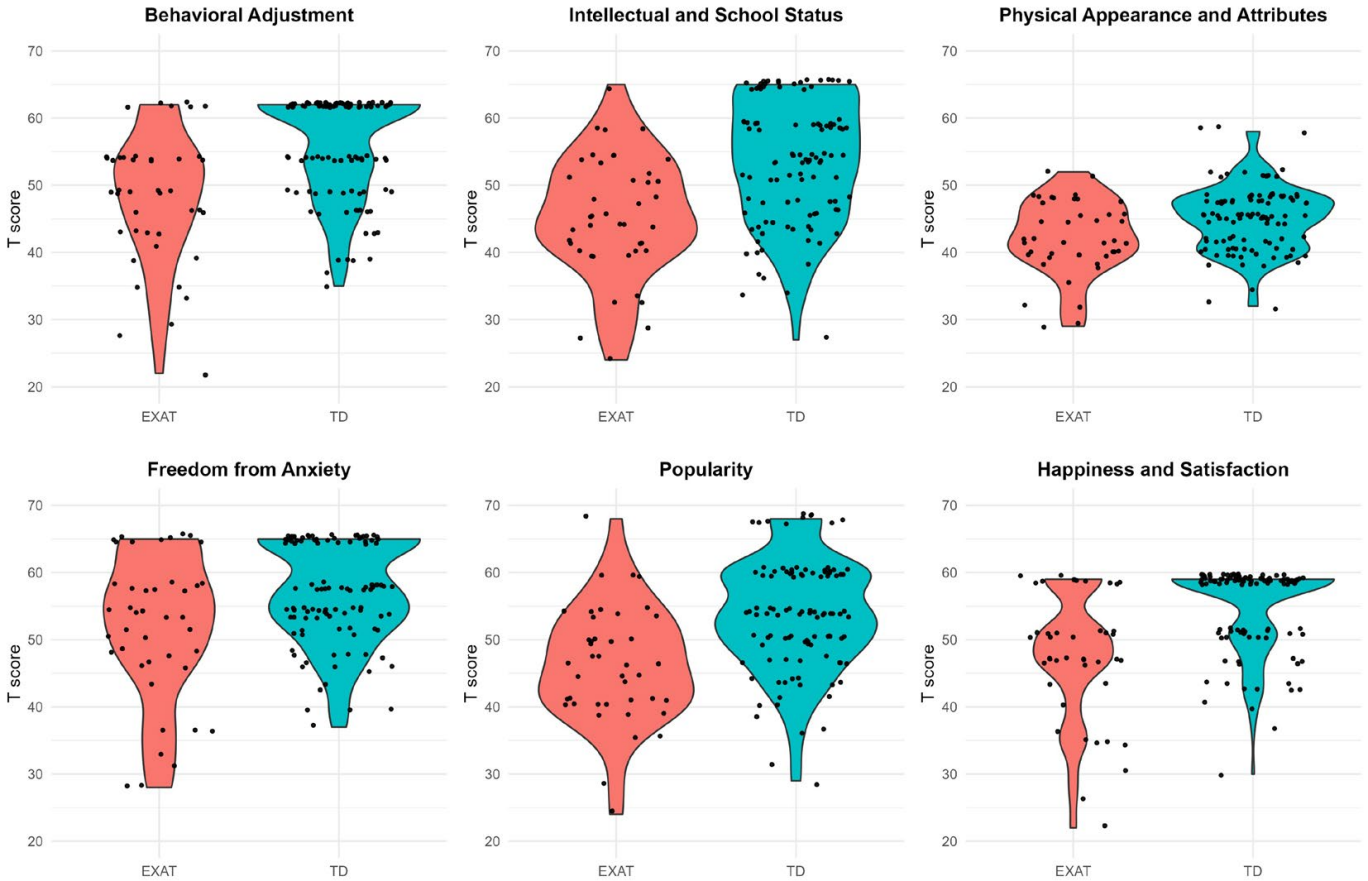
Differences and individual variation within the groups in self‐concept subscales (Piers‐Harris 2) in the participants referred for intervention (EXAT group) and typically developing (TD) group.

Differences in SC and EF between the EXAT and TD groups were examined and the findings revealed significant group differences across all measures (Table 2). These group differences and individual variation within the groups are illustrated in Figure 1. Although the mean T‐scores in all SC and EF scales were within the normal range, the EXAT group scored significantly lower (*p* < .001) than the TD control group in all SC scales. They also scored higher on parent‐rated BRIEF indices and subscales, indicating more problems in SC and EF within the EXAT group. In the EXAT group, about 33%–38% of children exceeded the clinical cut‐off scores on EF indices and 26%–38% on subscales, compared with 2%–3% and 0%–10% in the TD group. In terms of SC, only 2.4% children in the EXAT group exceeded the normal range of the SC total score, compared with 27.9% in the TD group, indicating more positive SC than usual. Approximately 20% of the children in the EXAT group had SC scores exceeding the normal variance limit. In contrast, in the TD group, only about 3% of the children had SC total scores below this limit, with a maximum of about 6.7% falling below this limit in the subscales, indicating larger than usual challenges in SC. This suggests that children in the EXAT group have a more negative perception of themselves and their own capacities, strengths and weaknesses.

**TABLE 2:** Difference between participants referred for intervention (EXAT group) and typically developing (TD) groups in self‐concept and parent rated executive functioning.

Scales	EXAT group		TD group		*W* ^a^	Effect size
	Md	Range	Md	Range		
Self‐concept (Piers‐Harris 2)						
Behavioral Adjustment	49.0	22.0–62.0	62.0	35.0–62.0	1175.5***	.378
Intellectual and School Status	45.0	24.0–65.0	54.0	27.0–65.0	1167.5***	.366
Physical Appearance and Attributes	42.0	29.0–52.0	45.0	32.0–58.0	1598.0*	.214
Freedom from Anxiety	54.0	28.0–65.0	58.0	37.0–65.0	1605.0*	.212
Popularity	47.0	24.0–68.0	54.0	29.0–68.0	1164.5***	.369
Happiness and satisfaction	47.0	22.0–59.0	59.0	30.0–59.0	1143***	.407
Total self‐concept	47.0	21.0–61.0	56.0	35.0–69.0	1024.0***	.416
Executive functions (BRIEF Parent ratings)						
Inhibition	57.5	37.0–83.0	42.0	36.0–64.0	3700.5***	.544
Shift	54.5	36.0–88.0	41.5	36.0–66.0	3271.5***	.390
Emotional Control	56.0	35.0–80.0	45.0	36.0–83.0	3273.5***	.390
Initiate	54.0	41.0–82	46.0	35.0–72.0	3356.0***	.420
Working Memory	63.0	35.0–83	45.0	35.0–72.0	3482.5***	.465
Planning/Organizing	51.5	39.0–80	43.0	33.0–86.0	3450.0***	.467
Organization of materials	58.5	33.0–71	48.0	32.0–71.0	3253.5***	.383
Monitoring	59.0	39.0–78	44.0	28.0–69.0	3911.0***	.618
Behavioral Regulation Index (BRI)	58.5	36.0–86	43.0	35.0–74.0	3563.5***	.494
Metacognition Index (MI)	58.5	42.0–96	44.0	31.0–74.0	3720.0***	.564
Global Executive Composite (GEC)	58.0	40.0–81	43.0	32.0–74.0	3763.0***	.576

^a^ Mann–Whitney‐Wilcoxon test.

* *p* < .05.

*** *p* < .001.

The influence of gender and age on SC was further explored. It was found that, overall, boys scored lower in Total SC (*W* = 1613, *p* = .0007, *r* = 0.282), Behavioral Adjustment (*W* = 1619, *p* = .0004, *r* = 0.292), Happiness and Satisfaction (*W* = 1807, *p* = .004, *r* = 0.235), Intellect and School Status (*W* = 1538, *p* < .0001, *r* = 0.309), and Popularity (*W* = 1567.5, *p* = .0003, *r* = 0.300). There were no gender differences in Freedom of Anxiety or Physical Appearance and Attributes. A negative correlation between age and Behavioral Adjustment (rho = −.185, *p* = .025), Freedom from Anxiety (rho = −.251, *p* = .002), Happiness and Satisfaction (rho = −.175, *p* = .034), and Intellectual and School Status (rho = −.171, *p* = .039) was found.

Given the differences in gender distribution between the EXAT and control groups, further analysis was conducted to determine whether group differences in SC persisted after controlling for gender and age effects. It was discovered that differences in Total SC score (beta = 8.00, *p* < .0001) and all subdomains (Behavioral Adjustment beta = 7.01, *p* < .0001), Intellectual and School Status (beta = 7.06, *p* < .001), Happiness and Satisfaction (beta = 6.88, *p* < .0001), Freedom from Anxiety (beta = 5.15, *p* = .002), Physical Appearance and Attributes (beta = 2.6, *p* = .008) and Popularity (beta = 6.28, *p* < .0001) remained significant after controlling for age and gender.

Additionally, due to differences in the level of learning support at school received by children in EXAT and TD groups, an investigation into whether varying levels of support impacted SC was conducted. Kruskal–Wallis tests revealed that children receiving general support at school demonstrated higher SC than those receiving intensified and special support in Behavioral Adjustment (*H* (2, *n* = 146) = 20.75, *p* < .0001) and Intellectual and School Status (*H* (2, *n* = 146) = 21.2, *p* < .0001). Furthermore, children with general support differed from those with special support in Freedom from Anxiety (*H* (2, *n* = 146) = 7.15, *p* = .028), Happiness and Satisfaction (*H* (2, *n* = 146) = 14.59, *p* = .0007), Physical Appearance and Attributes (*H* (2, *n* = 146) = 8.47, *p* = .014), and Popularity (*H* (2, *n* = 146) = 13.90, *p* = .001), while no differences were observed between children with intensified support and the other groups in these domains.

### Relationship between EF and SC

**FIGURE 2:**
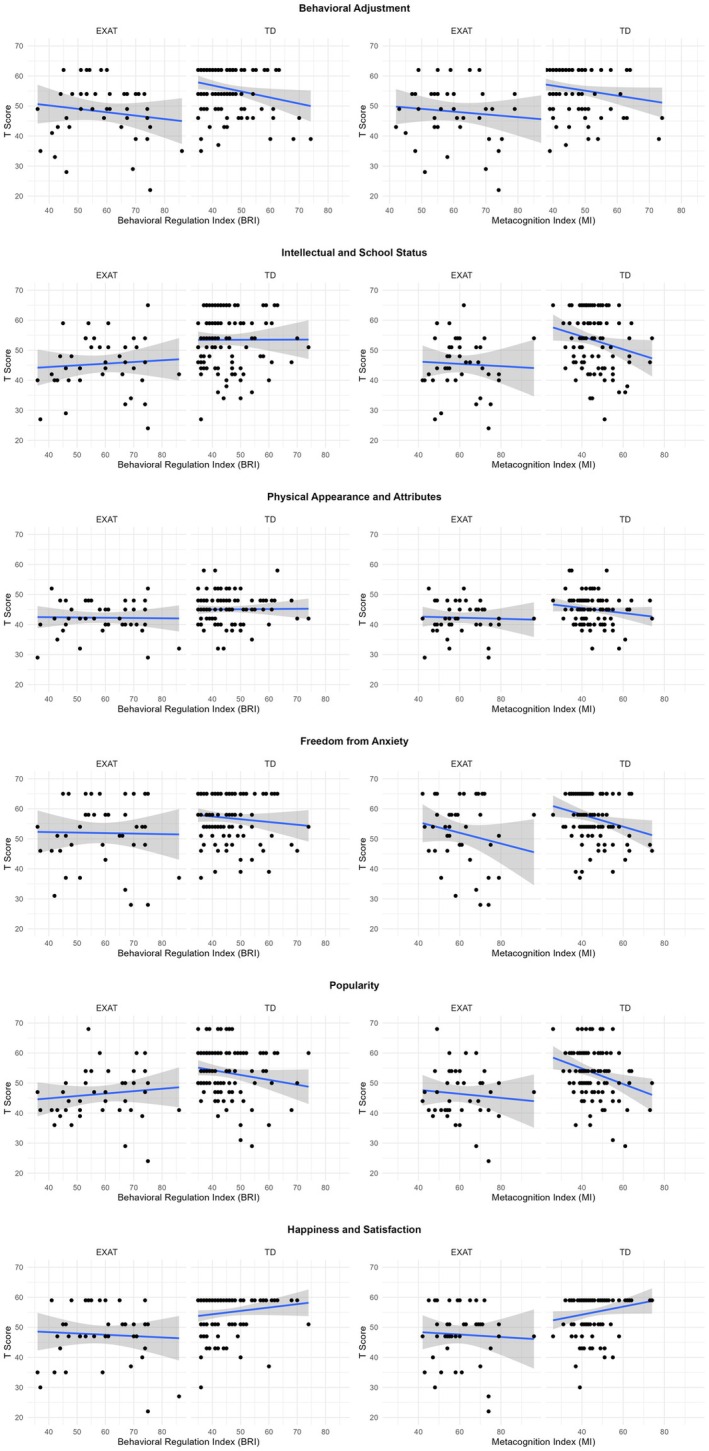
Relationship between self‐concept and executive functions for the participants referred for intervention (EXAT)) and typically developing (TD) groups separately.

The relationship between EF problems and SC was subsequently examined across all participants (Table 3, Figure 2). MI showed a negative correlation with SC in Behavioral Adjustment, Intellectual and School Status, Freedom from Anxiety and Popularity, suggesting that increased EF metacognition problems were linked to lower SC. BRI correlated negatively only with Behavioral Adjustment. Associations between SC subdomains and EF subscales were found, with Initiate, Working Memory and Plan/Organize linked to multiple SC subdomains. No associations between Shift and Organization of Materials with any SC subdomain were found. Among the SC subdomains, Behavioral Adjustment showed the most negative correlations with EF subscales. While SC subdomains generally correlated with EF subscales, Inhibition and Organization of Materials displayed fewer associations. SC related to Physical Appearance and Attributes and Happiness and Satisfaction were the only subdomains that showed no associations with EF subscales. The high correlations between GEC and SC were largely attributed to the substantial correlation between GEC and MI. Consequently, all subsequent analyses were conducted separately for MI and BRI.

**TABLE 3:** Correlations between self‐concept and executive functioning.

	1.	2.	3.	4.	5.	6.	7.	8.	9.	10.	11.	12.	13.	14.	15.	16.	17.
1. Inhibit																	
2. Shift	.60***																
3. Emotional Control	.67***	.77***															
4. Initiate	.47***	.60***	.60***														
5. Working Memory	.55***	.56***	.58***	.70***													
6. Plan/Organize	.49***	.59***	.62***	.72***	.75***												
7. Organization of Materials	.50***	.57***	.52***	.53***	.58***	.55***											
8. Monitor	.73***	.59***	.59***	.58***	.64***	.72***	.61***										
9. Behavioral Regulation Index (BRI)	.86***	.86***	.93***	.62***	.63***	.63***	.59***	.71***									
10. Metacognition Index (MI)	.61***	.65***	.65***	.82***	.86***	.87***	.70***	.78***	.71***								
11. Global Executive Composite (GEC)	.78***	.81***	.83***	.80***	.83***	.84***	.73***	.84***	.90***	.92***							
12. Behavioral Adjustment	−.42***	−.26	−.37*	−.29*	−.31*	−.34***	−.16	−.40***	−.36***	−.35***	−.39***						
14. Intellectual and School Status	−.14	−.20	−.18	−.26	−.28*	−.35***	−.14	−.29*	−.18	−.33***	−.28*	.61***					
14. Physical Appearance and Attributes	−.13	−.15	−.12	−.16	−.18	−.23	−.16	−.22	−.13	−.23	−.20	.35***	.62***				
15. Freedom from Anxiety	−.12	−.22	−.21	−.34***	−.30*	−.32***	−.20	−.25	−.19	−.32***	−.29*	.57***	.61***	.46***			
16. Popularity	−.22	−.23	−.23	−.31*	−.32***	−.37***	−.20	−.31*	−.24	−.37***	−.33***	.49***	.65***	.57***	.63***		
18. Happiness and Satisfaction	−.26	−.14	−.11	−.17	−.18	−.11	−.05	−.24	−.18	−.18	−.19	.67***	.53***	.55***	.57***	.48***	
19. Self‐concept Total	−.25	−.24	−.25	−.33***	−.35***	−.39***	−.18	−.35***	−.26	−.39***	−.36***	.77***	.88***	.68***	.80***	.78***	.70***

*Note*: All correlations were calculated using Spearman's rank correlation.

* *p* < .05.

*** *p* < .001.

### Group differences in relationship between EF and SC

Next, the group differences in associations between MI, BRI and SC were studied using hierarchical regression (Figure 2). The analyses revealed no significant interactions between group (EXAT and TD) and EF, as indicated in Tables S1–S7. However, a significant main effect of group was observed across all SC domains, consistent with previous analyses. When accounting for the group effect, MI showed a significant association with SC Total score, Freedom Anxiety and Popularity. Together, group and EF explained approximately 20% of the variance in SC, with the group's contribution ranging from about 5% to 17% depending on the SC domain. BRI was not significantly associated with any SC domain on its own.

Separate regression analyses for the groups showed that in the EXAT group, no significant associations were found between SC and EF. In contrast, in the TD group, EF difficulties (MI) were significantly associated with all SC domains except for Behavioral Adjustment and Happiness and Satisfaction. EF differences explained about 4%–7% of the variance in self‐concept in the TD group.

## DISCUSSION

This study examined the association between SC and EF in school‐aged children by comparing those referred for neuropsychological group rehabilitation (EXAT group, Rantanen et al., 2018) with typically developing peers (TD group). It extends prior research by examining overall SC and multiple domains beyond academic SC (Bailey et al., 2018).

The results show that the EXAT group scored lower in overall SC and all its domains compared with the TD group, despite SC mean scores being within the normative range. Only 2.4% of children in the EXAT group exceeded the normal SC range, compared with 27.9% in the TD group, suggesting a markedly less positive SC among children with EF difficulties. These findings align with previous evidence indicating that EF and attention difficulties are associated with a more negative SC, particularly in academic and emotional domains (Bailey et al., 2018; Betancourt et al., 2024; Capelatto et al., 2014; Pittari & Brown, 2020). Moreover, the results suggest that even subclinical EF difficulties may influence how children perceive their abilities and navigate peer relationships.

Across the entire sample, EF difficulties were consistently associated with a more negative SC, indicating that challenges in EF tend to co‐occur with less favourable self‐perceptions. However, contrary to expectations, this association emerged only among TD children and not among those referred for neuropsychological intervention, despite the latter group showing overall lower SC levels. Specifically, metacognition was related to nearly all SC domains in TD children, whereas no such associations were observed in the EXAT group. These unexpected findings challenge earlier assumptions that clinically significant EF problems would exert a stronger impact on self‐perception (Capelatto et al., 2014; Jacobs et al., 2002; Wigfield et al., 1997). Metacognition explained a meaningful share of variance in SC, while group membership (EXAT vs. TD) contributed a non‐trivial proportion that varied by domain. In contrast, the Behavioral Regulation Index was not independently associated with any SC domain. This pattern suggests that metacognitive skills, which support self‐reflection and self‐evaluation, may play a more central role in shaping self‐concept than behavioural regulation skills.

The present findings underscore that the links between EF and SC are multifaceted. EF difficulties, particularly in metacognition, may impair a child's ability to monitor and evaluate their own performance. This can disrupt daily activities, such as schoolwork, leading to repeated failures and negative feedback from the environment, which in turn diminishes SC and self‐esteem. Prior research indicates that EF deficits undermine SC via academic challenges (Cortés Pascual et al., 2019; Spiegel et al., 2021) and social difficulties (Holmes et al., 2016; Lonigan et al., 2017; Wang & Feng, 2024). However, some suggest that while reciprocal developmental associations are reported between self‐esteem, ADHD symptoms and peer relationships, peer problems did not mediate the links between later self‐esteem and ADHD symptoms (Russell et al., 2025). Key EF components—task organization, sustained attention and impulse control—are critical for success in both academic and social settings. These challenges may create a negative feedback loop: EF problems exacerbate a negative self‐concept, which in turn further impairs EF (Betancourt et al., 2024; Capelatto et al., 2014; Dapp & Roebers, 2021; Kita & Inoue, 2017; Safren, 2006).

Another potential explanation for the observed SC differences between children referred for intervention and TD peers is the positive illusory bias, which has been reported in children with ADHD (Hoza et al., 2002, 2004; Owens et al., 2007). It has been hypothesized that the illusory bias could serve as a coping mechanism that protects children's SC (Hoza et al., 2002, 2004). This hypothesis has later been supported in studies showing that children with ADHD symptoms regard themselves as having good social abilities and average SC, comparable to children with weak social abilities without ADHD symptoms (Capodieci et al., 2019). While such a positive bias may help maintain motivation (McQuade et al., 2017), it can hinder self‐awareness and responsiveness to feedback (Bailey et al., 2018). However, our study did not include objective performance measures or parent/teacher ratings related to children's self‐perceptions and therefore cannot confirm whether this bias explains the observed SC differences. Moreover, only about 36% of children in the EXAT group had an ADHD diagnosis, making this tendency an unlikely primary explanation for the overall pattern. Instead, the more negative SC among children referred for intervention likely reflects other factors, such as severity of difficulties and cumulative negative academic and social experiences.

Several factors may explain why EF–SC associations appeared only in TD children. The heterogeneity within the EXAT group, along with variations in EF difficulty type and severity, may have weakened detectable associations. Additionally, methodological considerations, such as reduced statistical power in subgroup analyses, could further obscure these relationships. Support from schools and families could buffer negative effects on SC (Savolainen et al., 2018). Cognitive awareness presents another layer of complexity: TD children may recognize minor EF challenges as deviations from peers, whereas children with EF deficits may ignore their difficulties due to underdeveloped EF and lack of insight into their own deficits or limited self‐awareness (Crisci et al., 2022; Fogel, 2022). However, beyond these explanations, the complex relationship between EF and SC likely involves various moderators and mediators that warrant further investigation. It is essential to explore how these factors differ between children with executive dysfunction and their typically developing peers (e.g. see Betancourt et al., 2024). Potential moderators, such as age, gender and school context, may influence the strength of the associations between EF and SC. Simultaneously, mediators like academic achievement, teacher feedback and emotion regulation could elucidate the mechanisms through which EF difficulties impact SC outcomes. Thus, a comprehensive examination of these moderators and mediators is crucial to fully understand the dynamics of the EF–SC relationship.

Beyond cognitive factors, contextual and demographic variables also shape SC. In the school context, EF difficulties often coincide with a greater need for learning support (Núñez et al., 2024), a pattern reflected in the present data. In the present study, higher levels of school support, indicative of more pervasive or severe difficulties, were associated with more negative SC in behavioural adjustment and academic domains compared with general support. SC also varies by grade level, gender and special educational needs status (DeVries et al., 2022; Savolainen et al., 2018). These findings underscore the importance of fostering a positive self‐image as part of learning support, aligning with prior evidence that SC plays a significant role in educational experiences.

Despite group‐level differences, considerable individual variability in SC was observed. Most children referred for intervention fell within the normative range, with only a minority showing lower‐than‐typical levels, whereas such deviations were rare among TD children. This pattern indicates that EF difficulties do not uniformly lead to negative SC, emphasizing the need for individualized assessment and support that address both EF and SC to prevent self‐reinforcing cycles of underachievement and negative self‐appraisal.

Gender and age also contributed to variation in SC. Girls reported more positive SC across domains, including academic and behavioural adjustment, while boys scored lower overall—a pattern consistent with international assessments, such as PISA (Programme for International Student Assessment), which report higher school satisfaction and achievement expectations among Finnish girls (OECD, 2023a, 2023b). These differences have been linked to factors, such as reading fluency, mastery‐oriented behaviour and homework engagement, whereas boys' performance is more strongly associated with parental education and socio‐economic status.

SC also showed a negative association with age, aligning with previous research suggesting that self‐concept tends to decline towards adolescence (Harter, 2012; Pesu et al., 2018). As children grow older and their ability to compare themselves with peers improves, academic and social challenges may increasingly undermine SC. Although this study was cross‐sectional and reflects age‐related differences rather than developmental changes, the pattern is consistent with evidence that academic performance and feedback shape SC from early on (Viljaranta et al., 2014). These patterns highlight the need for age‐ and gender‐sensitive approaches when supporting SC in educational settings.

### Limitations

This study can be considered representative of school‐aged children referred for neuropsychological rehabilitation due to clinically identified EF deficits. An advantage of the sample is that children had undergone neuropsychological assessments in which EF problems were judged to be significant and disruptive to everyday life, including schoolwork. Such clinical groups are essential, as most SC research focuses on typically developing children. The topic is important not only for long‐term prognosis but also for the development of clinical work and interventions.

Despite these strengths, several limitations should be noted. The clinical sample was relatively small, drawn from a single university psychology clinic providing rehabilitation services, and participants were not originally recruited for research purposes; the TD comparison group was likewise limited. These features may bias representativeness and constrain generalizability. The gender distribution also differed between groups, which warrants caution when interpreting gender effects.

Self‐reports are the most used instruments for measuring SC. In this study, we used the Piers–Harris 2, a widely recognized tool known for its strong internal consistency in normative samples (Piers & Herzberg, 2002) and other demographically representative groups (Guerin & Tatlow‐Golden, 2019). However, its psychometric properties can vary across populations, highlighting the importance of context in its application and interpretation. Studies involving children with EF deficits, ADHD or other neurodevelopmental conditions often overlook the reliability of SC scales, including the P‐H2 (Rizzo et al., 2010). In our sample, internal consistency for the overall SC score was good, but some subdomains showed notably lower reliability, raising concerns about the tool's applicability for children with EF difficulties.

The P‐H2 has also faced criticism regarding its psychometric properties. While it is user‐friendly and quick to administer, its suitability for children with EF difficulties/challenges and impulsivity can be questioned. A broader issue is the potential discrepancy between self‐reports and observer reports. For example, among college students with ADHD, self‐reported SC did not indicate problems while parents perceived significant difficulties (Nelson, 2011). This suggests that self‐assessments may not fully capture the complexities of SC in this population. In our sample, we did not find a systematic positivity bias in SC self‐evaluations among children with EF deficits. Nonetheless, future research should further validate the effectiveness and reliability of the P‐H2 in clinical populations experiencing neurobehavioural challenges.

A major limitation is reliance on parent‐reported questionnaires as the sole measure of EF. While behavioural ratings provide valuable insights into everyday functioning, they are vulnerable to parental bias and may not capture the multifaceted nature of EF across contexts (Sonuga‐Barke et al., 2013). Neuropsychological tests offer objective data on cognitive performance but may not reflect real‐world behaviour (Toplak et al., 2013). Parent report was selected for practical reasons and theoretical relevance, given that SC is shaped by children's daily experiences and parental perceptions. EF and attention deficits were confirmed at referral; however, only the clinician's summary was accessible, preventing direct comparison with parent ratings. Future studies should adopt multi‐informant, multi‐method approaches, integrating behavioural ratings with neuropsychological assessments to provide a more comprehensive picture of EF–SC links.

## CONCLUSIONS

The influence of EF on SC is evident, yet the relationships are complex and shaped by multiple mechanisms and contextual factors. Our findings highlight the importance of metacognitive processes for SC among typically developing children. They also raise questions about how clinical heterogeneity, compensatory support and measurement issues may weaken associations in referred populations. Further research is needed, particularly on the developmental trajectories of clinical groups facing EF difficulties. Identifying mediators related to EF and SC can help inform effective strategies to support and enhance self‐concept in children. Additionally, parental beliefs play a crucial role in shaping SC (Gniewosz et al., 2012; Simpkins et al., 2012), making parental involvement essential in interventions.

Clinically, early identification of EF difficulties is essential to prevent cascading effects on SC and academic performance. Practitioners should consider the complex links between EF and SC when designing interventions. Effective programmes should address cognitive deficits while actively fostering positive self‐perceptions, including metacognitive awareness, realistic self‐evaluation and constructive feedback practices. This dual approach can help break negative cycles of failure and low self‐esteem associated with EF challenges. Involving parents can further reinforce positive beliefs and motivation, creating a supportive environment for both learning and socioemotional development.

Future research should clarify the mechanisms linking EF and SC, including potential reciprocal effects (Guay et al., 2003). It should also examine moderators, such as age, gender and school context, as well as mediators like academic achievement, motivation, social competence, teacher feedback and emotion regulation to better understand EF–SC dynamics. Longitudinal studies are needed to track developmental trajectories and assess whether improvements in EF lead to sustained SC gains. Larger samples and randomized trials should evaluate combined interventions targeting both EF and SC while considering contextual factors, such as parental involvement and school support.

## AUTHOR CONTRIBUTIONS

**Elina Vierikko:** Conceptualization; methodology; data curation; investigation; formal analysis; writing – original draft; writing – review and editing; visualization; validation; resources. **Heini Saarimäki:** Methodology; data curation; formal analysis; writing – original draft; writing – review and editing; visualization; project administration. **Kati Rantanen:** Conceptualization; writing – review and editing; writing – original draft; project administration; data curation; methodology; investigation; validation; formal analysis; funding acquisition; resources.

## FUNDING INFORMATION

The authors received no funding for this study.

## CONFLICT OF INTEREST STATEMENT

The authors declare no conflicts of interest.

## Supporting information


**Table S1.** Hierarchical regression analysis for predicting SC Total score with group, EF subscales, and their interaction.
**Table S2.** Hierarchical regression analysis for predicting SC Behavioral Adjustment score with group, EF subscales, and their interaction.
**Table S2.** Hierarchical regression analysis for predicting SC Behavioral Adjustment score with group, EF subscales, and their interaction.
**Table S4.** Hierarchical regression analysis for predicting SC Intellectual and School Status score with group, EF subscales, and their interaction.
**Table S5.** Hierarchical regression analysis for predicting SC Physical Appearance and Attributes score with group, EF subscales, and their interaction.
**Table S6.** Hierarchical regression analysis for predicting SC Popularity score with group, EF subscales, and their interaction.
**Table S7.** Hierarchical regression analysis for predicting SC Happiness and Satisfaction score with group, EF subscales, and their interaction.

## Data Availability

Research data are not shared.
