# Supplementary material for: Relationships between executive functioning and self‐concept in children referred for neuropsychological group rehabilitation and typically developing children
Source: J Neuropsychol. 2026 Jan 14;20(2):375–93. doi: 10.1111/jnp.70029 (PMC13250377; doi:10.1111/jnp.70029)
Supplement: Supplementary file 1 — Table S1. Hierarchical regression analysis for predicting SC Total score with group, EF subscales, and their interaction. Table S2. Hierarchical regression analysis for predicting SC Behavioral Adjustment score with group, EF subscales, and their interaction. Table S2. Hierarchical regression analysis for predicting SC Behavioral Adjustment score with group, EF subscales, and their interaction. Table S4. Hierarchical regression analysis for predicting SC Intellectual and School Status score with group, EF subscales, and their interaction. Table S5. Hierarchical regression analysis for predicting SC Physical Appearance and Attributes score with group, EF subscales, and their interaction. Table S6. Hierarchical regression analysis for predicting SC Popularity score with group, EF subscales, and their interaction. Table S7. Hierarchical regression analysis for predicting SC Happiness and Satisfaction score with group, EF subscales, and their interaction. [file JNP-20-375-s001.docx]

**Table S1.**

*Hierarchical regression analysis for predicting SC Total score with group, EF subscales, and their interaction.*

| Self-concept TOTAL | Model 1 | Model 2 | Model 3 |
| --- | --- | --- | --- |
| Predictors | β | β | β |
| Group | 0.43 *** | 0.33 *** | 0.76 |
| EF BRI |  | 0.12 | 0.15 |
| EF MI |  | -0.28 * | 0.016 |
| Group x EF BRI |  |  | -0.0006 |
| Group x EF MI |  |  | -0.18 |
| 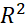 | 0.185 | 0.220 | 0.226 |
| 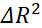 |  | 0.035 * | 0.006 |

Note. beta = standardized regression coefficient. * p < 0.05 ** p < 0.01 *** p< 0.001

**Table S2.**

*Hierarchical regression analysis for predicting SC Behavioral Adjustment score with group, EF subscales, and their interaction.*

| Self-concept Behavioral adjustment | Model 1 | Model 2 | Model 3 |
| --- | --- | --- | --- |
| Predictors | β | β | β |
| Group | 0.39 *** | 0.26 ** | 0.52 |
| EF BRI |  | -0.16 | -0.03 |
| EF MI |  | -0.08 * | -0.0096 |
| Group x EF BRI |  |  | -0.003 |
| Group x EF MI |  |  | -0.04 |
| 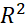 | 0.155 | 0.189 | 0.191 |
| 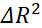 |  | 0.034 | 0.002 |

Note. beta = standardized regression coefficient. * p < 0.05 ** p < 0.01 *** p< 0.001

**Table S3.**

*Hierarchical regression analysis for predicting SC Freedom from Anxiety score with group, EF subscales, and their interaction.*

| Self-concept Freedom from Anxiety | Model 1 | Model 2 | Model 3 |
| --- | --- | --- | --- |
| Predictors | β | β | β |
| Group | 0.26 ** | 0.13 | 0.24 |
| EF BRI |  | 0.10 | -0.18 |
| EF MI |  | -0.32 ** | -0.31 |
| Group x EF BRI |  |  | -0.002 |
| Group x EF MI |  |  | -0.003 |
| 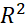 | 0.067 | 0.116 | 0.116 |
| 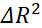 |  | 0.049 * | 0.0005 |

Note. beta = standardized regression coefficient. * p < 0.05 ** p < 0.01 *** p< 0.001

**Table S4.**

*Hierarchical regression analysis for predicting SC Intellectual and School Status score with group, EF subscales, and their interaction.*

| Self-concept Intellectual and School Status | Model 1 | Model 2 | Model 3 |
| --- | --- | --- | --- |
| Predictors | β | β | β |
| Group | 0.37 *** | 0.31 ** | 0.63 |
| EF BRI |  | 0.20 | -0.03 |
| EF MI |  | -0.29 * | 0.19 |
| Group x EF BRI |  |  | 0.006 |
| Group x EF MI |  |  | -0.30 |
| 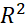 | 0.137 | 0.175 | 0.185 |
| 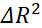 |  | 0.038 * | 0.010 |

Note. beta = standardized regression coefficient. * p < 0.05 ** p < 0.01 *** p< 0.001

**Table S5.**

*Hierarchical regression analysis for predicting SC Physical Appearance and Attributes score with group, EF subscales, and their interaction.*

| Self-concept  Physical Appearance and Attributes | Model 1 | Model 2 | Model 3 |
| --- | --- | --- | --- |
| Predictors | β | β | β |
| Group | 0.24 ** | 0.19 | 0.32 |
| EF BRI |  | 0.11 | -0.22 |
| EF MI |  | -0.19 | 0.24 |
| Group x EF BRI |  |  | 0.009 |
| Group x EF MI |  |  | -0.28 |
| 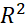 | 0.059 | 0.075 | 0.085 |
| 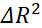 |  | 0.017 | 0.010 |

Note. beta = standardized regression coefficient. * p < 0.05 ** p < 0.01 *** p< 0.001

**Table S6.**

*Hierarchical regression analysis for predicting SC Popularity score with group, EF subscales, and their interaction.*

| Self-concept  Popularity | Model 1 | Model 2 | Model 3 |
| --- | --- | --- | --- |
| Predictors | β | β | β |
| Group | 0.36 *** | 0.24 * | 0.93 * |
| EF BRI |  | 0.11 | 0.38 |
| EF MI |  | -0.30 * | -0.06 |
| Group x EF BRI |  |  | -0.007 |
| Group x EF MI |  |  | -0.14 |
| 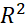 | 0.129 | 0.172 | 0.186 |
| 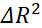 |  | 0.043 * | 0.014 |

Note. beta = standardized regression coefficient. * p < 0.05 ** p < 0.01 *** p< 0.001

**Table S7.**

*Hierarchical regression analysis for predicting SC Happiness and Satisfaction score with group, EF subscales, and their interaction.*

| Self-concept  Happiness and Satisfaction | Model 1 | Model 2 | Model 3 |
| --- | --- | --- | --- |
| Predictors | β | β | β |
| Group | 0.42 *** | 0.47 *** | -0.15 |
| EF BRI |  | 0.01 | -0.14 |
| EF MI |  | 0.09 | -0.23 |
| Group x EF BRI |  |  | 0.004 |
| Group x EF MI |  |  | 0.19 |
| 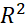 | 0.171 | 0.177 | 0.188 |
| 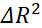 |  | 0.005 | 0.012 |

Note. beta = standardized regression coefficient. * p < 0.05 ** p < 0.01 *** p< 0.001
